# M. tuberculosis Gene Expression during Transition to the "Non-Culturable" State

**Published:** 2009-07

**Authors:** E.G. Salina, H.J. Mollenkopf, S.H.E. Kaufmann, A.S. Kaprelyants

**Affiliations:** 1 A.N. Bach Institute of Biochemistry, RAS;; 2 Max Planck Institute for Infection Biology

## Abstract

We analyzed the gene expression profile under specific conditions during reversible transition of M. tuberculosis cells to the "non-culturable" (NC) state in a prolonged stationary phase. More than 500 genes were differentially regulated, while 238 genes were upregulated over all time points during NC cell formation. Approximately a quarter of these upregulated genes belong to insertion and phage sequences indicating a possible high intensity of genome modification processes taking place under transition to the NC state. Besides the high proportion of hypothetical/conserved hypothetical genes in the cohort of upregulated genes, there was a significant number of genes belonging to intermediary metabolism, respiration, information pathways, cell wall and cell processes, and genes encoding regulatory proteins. We conclude that NC cell formation is an active process involved in the regulation of many genes of different pathways. A more detailed analysis of the experimental data will help to understand the precise molecular mechanisms of dormancy/latency/persistence of M. tuberculosis in the future. The list of upregulated genes obtained in this study includes many genes found to be upregulated in other models of M. tuberculosis persistence. Thirteen upregulated genes, which are common for different models, can be considered as potential targets for the development of new anti-tuberculosis drugs directed mainly against latent tuberculosis.

## INTRODUCTION

Mycobacterium tuberculosis – the causative agent of tuberculosis – can persist in the human host for decades after infection. Such a latent M. tuberculosis state is traditionally connected with its transition to the dormant state, accompanied by loss of culturability [1]. This makes it practically impossible to reveal latent infection by traditional biochemical and microbiological means and attempt to cure it by antibiotic therapy. To study latent infection in live organisms, several modifications of the experimental model of dormancy during hypoxia in vitro are used [2, 3]. However, none of them imitates such an important state of bacteria as their "non-culturability" in the dormant state. We have established an experimental model where dormant M. tuberculosis cells are "non-culturable" (NC) and can reactivate under special conditions [4].

To reveal the biochemical processes accompanying the transition of bacteria to the NC state and to understand the mechanisms of this phenomenon, we analyzed M. tuberculosis gene expression profile during the formation of NC cells.

## Methods

M. tuberculosis total RNA samples were extracted from cells in the late logarithmic phase (5 days of cultivation) and during the transition of cells to the NC state under incubation in the stationary phase at different time points (21, 30, 41 and 62 days of cultivation) as described previously [5]. cDNA was generated from 1µg RNA using random hexamers and reverse transcriptase (Superscript III, Invitrogen, Karlsruhe, Germany) according to the manufacturer’s instructions. Reverse transcribed samples were purified with the QIAquick PCR purification kit (Qiagen, Hilden, Germany) and labeled with Cy3- and Cy5-ULS according the suppliers' recommendations (Kreatech Diagnostics, Amsterdam, The Netherlands). Finally, labeled samples were purified with KRE Apure spin columns. Microarray experiments were performed as dual-color hybridizations. In order to compensate for the specific effects of the dyes and to ensure statistically relevant data, a color-swap dye-reversal analysis was performed. Cy3-labeled cDNA (250ng) corresponding to cells from different time points in the stationary phase was competitively hybridized with the same amount of Cy5-labeled cDNA of the control sample as color-swap technical replicates onto self-printed microarrays comprising a collection of 4,325 M. tuberculosis-specific "Array-Ready" 70mer DNA oligonucleotide capture probes and 25 control sequences (Operon Biotechnologies, Koeln, Germany) at 42°C for 20 h. Arrays were washed 3 times using a SSC wash protocol followed by scanning at 10 µm (Microarray Scanner BA, Agilent, Technologies, Waldbronn, Germany). Image analysis was carried out with Agilent’s feature extraction software version (Agilent, Technologies, Waldbronn, Germany). The extracted MAGE-ML files were further analyzed with the Rosetta Resolver Biosoftware, Build 7.1 (Rosetta Biosoftware, Seattle, USA). Ratio profiles comprising color-swap hybridizations were combined in an error-weighted fashion to create ratio experiments. Anticorrelation of dye-reversals was determined by the compare function of Resolver. Next we applied a Student's t-test. Finally, by combining a 1.5-fold change cutoff to ratio experiments and the anticorrelation criterion together with the signatures from the Student's t-test, all valid data points had a P-value < 0.01, rendering the analysis highly robust and reproducible.

## Results and discussion

**Fig. 1.**
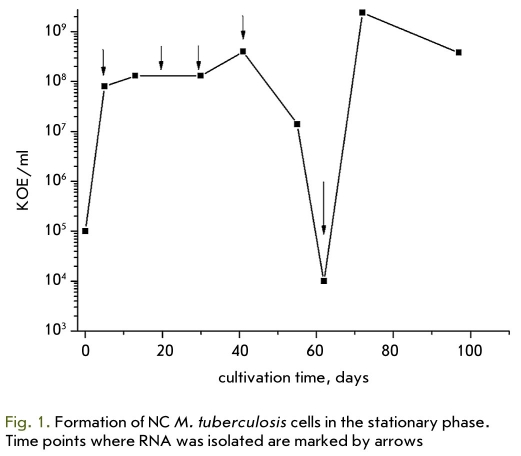
Formation of NC M. tuberculosis cells in the stationary phase. Time points where RNA was isolated are marked by arrows

We found earlier that M. tuberculosis cultivation in the modified Sauton medium without K+ supplemented by dextrose, BSA, and sodium chloride led to a decrease in colony forming units (CFU) on the solid medium in the stationary phase [4]. After 60 days of cultivation, the CFU count dropped to 105 per ml (Fig. 1), which meant a transition of 99.9% of cells to the NC state. During further cultivation of cells, spontaneous recovery of NC cells was observed. It is important that the NC state was reversible, and that cells with a minimum CFU count could be reactivated after regrowing them in fresh medium.

Comparison of the gene expression profile at different time points from the stationary to the logarithmic phase (5-day cultivation) revealed a different expression (at least 1.5-fold) for a significant number of genes (566), which corresponds to 14% of the M. tuberculosis genome. Some 238 genes are upregulated and 237 downregulated over all time points during the entire culture period. Table 1 shows the functional category of differentially regulated genes during the transition of cells to the NC state.

**Table 1:** Functional categories of M. tuberculosis genes with changed expression level during transition to the NC state

Functional categories	Genes induced during transition to the NC state		Genes repressed during transition to the NC state		Percent (%) in the genome
	Number of genes	%	Number of genes	%	
Virulence, detoxification, adaptation	5	2.1	7	2.9	2.6
Lipid metabolism	6	2.5	20	8.4	5.9
Information pathways	13	5.5	23	9.7	5.8
Cell wall and cell processes	24	10.1	59	24.8	18.8
Insertion sequences and fages	58	24.4	1	0.4	3.7
PE/PPE	7	2.9	11	4.6	4.2
Intermediary metabolism and respiration	42	17.7	50	21.1	22.4
Regulatory proteins	16	6.7	4	1.7	4.8
Unknown/hypothetical	67	28.1	63	26.5	31.9
Total number of genes	238	–	237	–	3924

Besides the significant amount of conserved hypotheticals/ unknown genes, many genes involved in the intermediary metabolism and respiration, virulence, detoxification and adaptation, lipid metabolism, information pathways, cell wall and cell processes were downregulated. A considerable amount of genes coding hypothetical proteins were also found to be upregulated in the NC state: remarkably, genes encoding insertion sequences and phages represented about a quarter of the genes upregulated in the NC state, whereas their proportion in the genome was smaller – only 3.7%. This fact is a possible illustration of the high intensity of genome modification processes during the transition of cells to the NC state.

A significant proportion of upregulated genes belonged to the intermediary metabolism and respiration category, in particular, gcvB and ald, coding, respectively, glycine dehydrogenase and L-alanine dehydrogenase, proteases pepR and clpC2. icl1 – one of the genes coding isocitrate lyase, anaplerotic enzyme, existing in the M. tuberculosis cells in two isoforms icl 1 and icl 2 – was found upregulated. Isocitrate lyase is the key enzyme of the glyoxylate cycle – a metabolic pathway, which is an alternative for the tricarboxylic acid cycle and allows the synthesis of carbohydrates from simple precursors. In particular, it plays an important role in seed germination, where fatty acids are used as the main storage of carbon and energy. The induction of some genes involved in lipid degradation, such as fadD9, fadE24, fadE26, and fatty acid degradation, scoA, is indicative of the active role of the glyoxylate cycle in NC cells already found for the Wayne persistence model [2].

During transition to the NC state, some genes used as markers of stress conditions were induced: the heat-shock protein hsp, the chaperones Rv0440 and Rv3417с, as well as sigma-factors: sigG – regulating genes which are necessary for survival inside the macrophages and sigB, which can control stationary phase regulons and general resistance to stress. Induction of ccsA, whose product takes part in the cytochrome biosynthesis at the step of heme attachment, and cyp132, coding one of the cytochrome’s P450 oxidizing different xenobiotics, could evidently reflect accumulation of toxic components in cultures during transition. Enzymes of the non-mevalonate pathway of isoprenoid biosynthesis ispF and ispD were also induced in the NC cells. There are data indicating that some of the metabolites of this pathway can affect the immune response of the host [6]. A number of induced genes are involved in the information pathways and those encoding regulatory proteins; in particular, the transcriptional regulator furA, which acts as a global negative control element, employing Fe2+ as a cofactor to bind the operator of the repressed genes. It seems to regulate the transcription of katG, which is induced in the NC state. katG encodes a multifunctional enzyme, exhibiting both catalase, a broad-spectrum peroxidase and peroxynitritase activities and is believed to play a role in the intracellular survival of mycobacteria within macrophages, protecting them against the aggressive components produced by phagocytic cells. Some genes taking part in the cell wall and cell processes, in particular the transporters ctpG and ctpC encoding atpases-transporting metal cations and the transporter Rv2688с involved in antibiotic resistance and export of antibiotics across the membrane, are activated.

To identify the genes that were significantly upregulated during transition to the NC state, we used stringent criteria: the expression level during the whole time course in the stationary phase was upregulated at least 3-fold in comparison to the expression in the logarithmic phase. Fifty-one genes met this criterion Table 2.

**Table 2:** Significantly up-regulated genes during transition to the NC state in the stationary phase

ORF	Gene	Gene product	Changing of gene expression level				
			5 days	21 days	30 days	41 days	62 days
Rv0186	bglS	Beta-glucosidase	1	4.20459	8.33686	6.51867	5.24295
Rv0840c	pip	Proline iminipeptidase	1	6.33559	11.0004	4.58881	3.86572
Rv0841c		Transmembrane protein	1	31.11093	52.56174	13.79488	11.85425
Rv0989c	grcC2	Polyprenil-diphosphate synthase	1	7.60797	6.29748	7.58723	3.94285
Rv0990c		Hypothetical protein	1	7.12899	6.60915	6.652	3.57343
Rv0991c		Conserved hypothetical protein	1	3.31598	3.87521	5.44297	3.70462
Rv1369c		Transposase	1	3.17178	3.9213	4.22925	3.86883
Rv1394c	cyp132	Cytochrome P450 132	1	8.89047	7.50161	3.72981	3.12534
Rv1395		Transcriptional regulatory protein	1	3.22394	11.65875	7.03908	4.27327
Rv1397c		Conserved hypothetical protein	1	6.95276	11.79184	5.97336	5.77752
Rv1460		Transcriptional regulatory protein	1	3.87617	5.50637	6.90405	3.78332
Rv1575		phiRV1 phage protein	1	17.29509	37.08693	51.7473	20.53329
Rv1576c		phiRV1 phage protein	1	28.17817	33.97652	10.11378	12.88182
Rv1577c		phiRV1 phage protein	1	26.27261	39.87495	19.41512	11.49041
Rv1584c		phiRV1 phage protein	1	3.27674	5.68552	3.3055	3.02886
Rv1831		Hypothetical protein	1	3.1468	5.74692	5.14019	4.04747
Rv1991c		Conserved hypothetical protein	1	4.04696	4.12618	4.06786	4.65579
Rv1992c	ctpG	Metal cation transporter ATPase	1	5.2883	7.31348	4.7442	4.22806
Rv2106		Transposase	1	3.01418	5.61324	4.77882	5.09925
Rv2254c		Integral membrane protein	1	7.09534	6.53956	3.33899	4.63885
Rv2278		Transposase	1	3.28663	6.78129	6.28036	4.13102
Rv2354		Transposase	1	3.1594	6.15299	5.21098	3.13151
Rv2497c	pdhA	Pyruvate dehydrogenase alpha subunit	1	3.73133	4.52197	5.04976	4.00306
Rv2642		ArsR family transcriptional regulator	1	3.76985	5.16757	4.39006	3.93426
Rv2644c		Hypothetical protein	1	3.36059	7.58921	5.36796	3.51825
Rv2645		Hypothetical protein	1	3.45006	8.21393	6.70101	3.25709
Rv2646		Integrase	1	5.04391	12.16535	7.82435	9.96087
Rv2647		Hypothetical protein	1	5.32983	13.40623	9.43796	7.2163
Rv2649		Transposase IS6110	1	3.2505	5.3557	5.59089	3.74714
Rv2650c		phiRv2 prophage protein	1	21.46669	29.74372	16.65359	20.66349
Rv2651c		phiRv2 prophage protease	1	20.04086	34.29153	20.61728	13.41666
Rv2660c		Hypothetical protein	1	13.43717	41.25793	67.29882	19.6699
Rv2661c		Hypothetical protein	1	9.23174	28.30861	52.34967	11.04351
Rv2662		Hypothetical protein	1	20.62942	18.83647	14.72059	12.88898
Rv2663		Hypothetical protein	1	7.61461	9.43216	8.19525	7.3034
Rv2664		Hypothetical protein	1	6.24636	8.49102	7.10191	5.60291
Rv2666		Truncated transposase IS1081	1	6.91867	13.89339	7.89331	5.86579
Rv2667	clpC2	ATP-dependent protease	1	9.44815	17.89662	9.64508	6.46149
Rv2707		Conserved transmembrane protein	1	3.35002	5.09024	14.83903	4.53239
Rv2711	ideR	Transcriptional regulatory protein	1	3.48877	4.30099	6.06795	3.83858
Rv2713	sthA	Soluble pyridine nucleotide transhydrogenase	1	4.43327	6.35516	6.80833	3.83838
Rv2780	ald	Secreted L-alanine dehydrogenase ALD	1	5.2891	4.65988	4.52694	4.92656
Rv2814c		Transposase	1	3.3279	5.52338	4.86873	4.60102
Rv2815c		Transposase	1	3.13667	6.24306	5.87423	4.84337
Rv3185		Transposase	1	3.58899	6.43621	5.67335	5.82686
Rv3186		Transposase	1	3.2903	6.21375	6.14822	5.77427
Rv3290c	lat	L-lysine aminotransferase	1	4.32023	5.06387	3.54801	3.9704
Rv3474		Transposase IS6110	1	3.04947	6.19754	6.19869	3.22266
Rv3475		Transposase IS6110	1	3.73966	5.79892	5.63617	6.23465
Rv3580c	cysS	Cysteinyl-tRNA synthetase	1	3.87797	6.67899	3.14124	3.40852
Rv3582c	ispD	2-C-methyl-D-erythritol 4-phosphate cytidylyltransferase	1	3.50012	4.07861	3.78626	3.51221

Among the genes with a substantially high level of expression, genes encoding insertion sequences and phages – 20 genes out of the 51– are prime candidates, while 13 genes encode hypothetical proteins with unknown function. It is remarkable that the significantly upregulated genes belonged to intermediary metabolism and the respiration category. Moreover, these genes mainly encode proteins involved in degradation processes; namely bglS – beta-glycosidase (hydrolyzes the terminal, non-reducing beta-D-glucose residue); pip – proline iminopeptidase (specifically catalyses the removal of N-terminal proline residues from small peptides); clpC2 ATP-dependent protease; and ald – L-alanine dehydrogenase (catalyses alanine hydrolyze – an important constituent of the peptidoglycan layer). In addition, the pdhA coding the alpha subunit of pyruvate dehydrogenase and taking part in the energetic metabolism and catalyzing the conversion of pyruvate to acetyl-CoA was highly expressed. Significant upregulation of sthA, a soluble pyridine nucleotide transhydrogenase that catalyses the conversion of NADPH generated by catabolic pathways to NADH, which is oxidized by the respiratory chain for energy generation, is a sign of the prevalence of catabolic reactions in cell metabolism in the NC state.

Analysis of the global gene expression profile has been published for several M. tuberculosis persistence models, in particular the Wayne model of the non-replicating state during hypoxia [5,7,8], the gradual depletion of the carbon source under decreased oxygen tension [9], the adaptation of M. tuberculosis within macrophages [10], and in vivo within artificial granulomas in mice [11]. Considering the results of these studies, the gene expression profile in our model of "non-culturability" in the stationary phase has, evidently, some overlaps with the above-mentioned models of persistence Table 3.

**Table 3:** Comparison of genes upregulated during transition to the NC state in the stationary phase (at least 1.5-fold) to the genes activated in other models of persistence

Models of M. tuberculosis persistence	Overlapping to 238 genes activated in the stationary phase during transition to the NC state.	
	Number of genes	%
Wayne non-replicating state (Voskuil et al., 2004)	23	9,7
Persistence at gradual depletion of carbon source at 50% oxygen tension (Hampshire et al., 2004)	82	34,5
Persistence within macrofages (Schnappinger et al., 2003)	77	32,4
Artificial granuloma in mice (Karakousis et al., 2004)	32	13,4
Enduring hypoxia response (Rustad et al., 2008)	40	16,8

Little in common was found between the genes induced in our model of "non-culturability" and the Wayne dormancy model during hypoxia Table 3. The Wayne model is characterized by the induction of genes of the dormancy survival regulon (Dos-regulon), a group of 49 genes under the control of devR which codes the regulatory part of the two-component system. Upregulation of the Dos-regulon was found not only for dormant cells under hypoxia in vitro, but also for M. tuberculosis cells within macrophages [10], and in the artificial granulomas in mice [11]. In our model of M. tuberculosis transition to the NC state in the stationary phase, only two genes from Dos-regulon – Rv0571c and Rv2631 – were found upregulated. Dos-regulon induction was not found in the persisting cells during starvation [12], and only two genes of Dos-regulon were activated during persistence at gradual depletion of the carbon source [9].

A recently published paper [13] demonstrated that the role of Dos-regulon is apparently overestimated not only as a universal regulator of the dormant state of mycobacteria, but also as a general response on hypoxia. Genes of the Dosregulon were shown to be activated only 2 hours after hypoxia.

Thereafter expression of at least half of these returned to the baseline [13]. The authors observed a significant induction of another 230 genes after further cultivation during hypoxia, and hereafter their expression level was stable. Thus, the authors refer to this group of genes as enduring hypoxia response (EHR) genes. Considering the gene expression profile for our model of transition to the NC state, we found significant overlap with this group of genes Table 3, which was rather unexpected because the conditions for NC cell formation developed in our laboratory did not imply any oxygen limitation. Some overlap with EHR [13] was found for the persistence model of gradual depletion of the carbon source [9] and the transcriptional response to multiple stresses [14]. Therefore, it is possible to conclude that EHR genes may not only play a role as hypoxia markers, but may also be a general regulon of the dormant state of M. tuberculosis, independent of its induction.

Thus, the data presented here indicate that cell transition to dormant state is an active process and that numerous genes are involved in it. The future task is to investigate this process in detail in order to understand the molecular mechanisms in the cells during the transition to the dormant state.

Based on the results of the transcriptome analysis of the NC cells obtained in our model and those obtained in several models of persistence, it is possible to pinpoint some shared genes that are upregulated in these models Table 4. The genes presented in Table 4 and their products are believed to be important for further study, because some of them could represent new targets for anti-tuberculosis drug candidates directed mainly against latent tuberculosis.

**Table 4:** Shared genes of M. tuberculosis persistence state. Genes of EHR regulon are in bold

ORF	Gene	Non-replicating state of Wayne (Voskuil et al., 2004)	Gradual depletion of carbon source (Hampshire et al., 2004)	Persistence within macrophages (Schnappinger et al., 2003)	Artificial granuloma in mice (Karakousis et al., 2004).	NC state in the stationary phase (this study)
Rv0188		0.8	67.2	2.8	2.7	2.5
Rv0211	pckA	-	1.7	3.6	2.6	1.64
Rv0251c	hsp	4.5	18.6	25.6	3.9	4.5
Rv1894c		2.0	5.1	1.8	-	2.8
Rv1909c	furA	-	5.4	2.2	2.8	2.7
Rv2011c		2.1	9.5	2.5	-	2.8
Rv2497c	pdhA	3.4	8.4	2.1	2.0	4.0
Rv2660c		1.5	4.3	2.1	3.3	19.7
Rv2662		1.5	1.5	2.0	-	12. 9
Rv2710	sigB	-	34.6	3.8	4.7	4.6
Rv2780	ald	6.1	2.6	2.4	2.4	4.9
Rv3139	fadE24	-	2.2	2.0	5.8	2.4
Rv3290c	lat	3.6	25.9	7.5	5.6	4.0

## Acknowledgements

This work was supported by the Program of the Presidium of the RAS "Molecular and Cellular Biology"
